# (Un)Sustainable Science: Greening Practices in Research, Clinical Microbiology, and Veterinary Laboratories Locally and Globally

**DOI:** 10.1093/ofid/ofae677

**Published:** 2025-01-10

**Authors:** Bethel Alebel Bayrau, Esra Buyukcangaz, Sapna P Sadarangani, Bartholomew N Ondigo, Andrea Prinzi, A Desiree LaBeaud

**Affiliations:** Division of Infectious Diseases, Department of Pediatrics, Stanford University, Stanford, California, USA; Division of Infectious Diseases, Department of Pediatrics, Stanford University, Stanford, California, USA; Department of Microbiology, Faculty of Veterinary Medicine, Bursa Uludag University, Bursa, Turkiye; Department of Infectious Diseases, National Centre for Infectious Diseases, Singapore, Singapore; Department of Infectious Diseases, Tan Tock Seng Hospital, Singapore, Singapore; Lee Kong Chian School of Medicine, Nanyang Technological University, Singapore, Singapore; Department of Biochemistry and Molecular Biology, Egerton University, Njoro, Kenya; Medical Affairs, bioMérieux, Salt Lake City, Utah, USA; Division of Infectious Diseases, Department of Pediatrics, Stanford University, Stanford, California, USA

**Keywords:** diagnostic stewardship, global health, green laboratories, sustainability, waste

## Abstract

Health care, veterinary, and research facilities produce tremendous amounts of waste and account for a significant proportion of their institutions’ energy and water use. The majority of municipal solid waste produced by these facilities gets unsustainably disposed of, including exportation to lower-income countries, and most of the plastic waste is nonrecyclable and nondegradable. The produced waste not only results in excessive carbon emissions harming planetary health but also poses direct harm to human health, broadens global inequities, and produces avoidable economic costs. Greening up laboratories by reducing waste production and water and energy use offers many benefits and does not have to be time or resource intensive. Sustainable practices to green up laboratories include reusing materials, decreasing energy use by choosing low-energy settings and shutting off equipment when not in use, installing low-flow faucets to decrease water use, proper sorting of waste, environmentally conscious purchases of supplies, and avoiding unnecessary medical and veterinary tests.

## Teaser

Medical, research, and veterinary laboratories produce significant amounts of waste, causing massive carbon emissions. This article provides practices that institutions and laboratories can implement to green up their facilities, and it gives a global perspective on health care waste management.

Health care facilities and research laboratories produce a significant amount of waste each year, contributing to environmental and plastic pollution. Their tremendous waste production and unsustainable disposal of waste cause direct and indirect harm to human and planetary health, exacerbate global inequality with ongoing waste disposal in lower-income countries, produce excessive greenhouse gas (GHG) emissions, and result in high institutional costs that could be avoided [1]. Several reports have shown detrimental consequences of GHG emissions and climate change on infectious diseases specifically, with an unprecedented and significant increase in prevalence and transmission susceptibility, including wider geographic spread of climate-sensitive infectious diseases and risk for outbreaks and epidemics that can be life-threatening [2]. Laboratory practices within health care, veterinary medicine, and scientific research are responsible for a large part of the environmental footprint of institutions, accounting for the majority of their energy and water use. The health care system as a whole accounts for 4.4% of global net GHG emissions, with regional and country variations [3]. For example, health care accounts for 8% of total GHG emissions from the United States [1]. With limited material recycling capacity and facilities as well as the inability to recycle most plastic products, recycling efforts are insufficient to mitigate the detrimental effects of waste [4]. However, lowering waste production and energy/water utilization through sustainability efforts can be effective in reducing the environmental footprint of laboratories and offer tremendous cost and carbon savings, and they should be implemented by all institutions [5–7]. Small changes in practice can have important gains and do not have to be time or resource intensive, and they can yield other benefits, such as enhancing overall safety, work environment, and quality of research. In this article, we discuss such changes and practices that can green up research, medical, and veterinary laboratories, and we give a real-world global perspective from Kenya on health care waste management.

## GREENING RESEARCH LABORATORIES

In recent years, efforts to combat and study climate change have exponentially increased worldwide. However, it is important to raise awareness about research laboratories’ contribution to climate change and adopt practices that are eco-friendly to reduce GHG emissions and waste production. Each year, research laboratories produce 12 billion lb (5.5 million metric tons) of plastic waste, an estimated 1% to 2% of global plastic use [8]. As compared with office spaces of the same size, research laboratories use 4 times as much water as well as 5 to 10 times or even 100 times the amount of energy [6, 9]. Furthermore, −80 °C freezers alone use the energy equivalent of a single US household's energy, and fume hoods use the energy of 3.5 households when running at full speed [10, 11]. Moreover, it is estimated that research laboratories utilize up to 70% of their institution's energy but take up only 5% to 20% of the total space [9, 12–14]. A study in 2011 that compared 2 similar clinical trials found that intention matters: the GHG emissions of the trial without efforts to reduce emissions was 181 tons in 1 year, approximately 73 tons more than the clinical trial with efforts to reduce GHG emissions [15]. This study proves that with effort and by adopting eco-friendly practices, the carbon footprint of research practices can be significantly reduced. There are ample practices that can be utilized for greener research laboratories.

## Reducing Waste Production

One way to reduce contribution to GHG emissions is addressing the production and management of waste. There are hierarchies to waste management practices with different levels of environmental impact. Reducing or eliminating waste production offers the highest impact on sustainability and should be prioritized. Reducing waste production can be achieved by practicing stewardship, using materials on a necessity basis, implementing green procurement practices, and reusing materials where feasible, especially for wet laboratories that conduct experiments. Whenever possible, working with and purchasing materials that can be reused after sterilization, such as glassware, metal, and autoclavable plastics, can significantly reduce waste production—specifically, plastic waste that is nondegradable [16, 17]. The energy use during sterilization procedures needs to be considered in the decision-making process. Building professional habits, creating an institutional culture, and implementing institutional protocols for purchasing environmentally conscious laboratory items can also have a significant effect. Eco-friendly purchases include non–single-use items (eg, refillable pipette tip boxes) and alternatives to plastics, such as consumables made from metal, paper, or wood or products with reduced plastic content [18]. To aid in procurement processes, laboratory personnel can refer to the ACT database (Accountability, Consistency, and Transparency), which features an Environmental Impact Factor, a metric for the environmental impact of laboratory products [19]. Additionally, certain instruments and equipment can be shared within and across institutions.

Another practice to promote sustainability is recycling materials and waste. Some institutions and product companies offer recycling programs that laboratories can utilize, such as Fisher Scientific's Pipette Tip Box Recycling and Kimberly-Clark's RightCycle. Laboratories should also implement strategies to appropriately dispose of different types of waste and avoid contamination of materials with hazardous agents to ensure all materials are recycled as much as possible. It is important to note, however, that most plastic products are not recyclable and only a small fraction of recyclable materials actually get recycled. Therefore, though recycling waste is important, laboratories should prioritize reducing waste production at the source. Last, nonrecyclable plastic materials can be innovatively repurposed into art or other functions, such as pots for plants [20].

On the journey to making a habit of sustainable practices surrounding waste, having an awareness of each laboratory's waste production and management through a waste audit can be a beneficial starting point. A waste audit is a survey used to determine the amount and types of waste produced by an entity. It can also shed light on waste management practices and areas of improvement [21]. As many waste audit protocols exist, laboratories should choose and modify protocols that fit their operations best. One protocol developed by a research group from Stanford University involves collecting laboratory waste daily and documenting its weight, count, type, and disposal location at the end of each week over a 2-week period. Analyzing waste audit results and discussing their implications can enable laboratories to improve self-awareness regarding waste generation, waste management, and material usage. This process identifies opportunities for adopting eco-friendly practices and initiates self-reflection and dialogue across diverse stakeholders and the wider health care system to enable greener laboratory practice.

## Reducing Energy And Water Usage

In addition to reducing solid waste, laboratories should reduce energy and water usage. Installing low-flow faucets, automatic shut-off valves, and foot pedals allows for easy turning on and off and can significantly reduce water consumption. Installing low-flow aerators can reduce flow to <1.5 gallons/min from typical taps at 4 gallons/min [22]. UC San Diego has saved 900 000 gallons of water per year after installing aerators in laboratory buildings [22]. Substituting tap water for deionized water whenever possible can also reduce water usage, as for each liter of deionized water produced, 3 L of tap water is used [18].

Most energy use in research laboratories is related to appliances for air circulation and cold storage [23]. Laboratories can increase the temperature of their −80 °C freezers to −70 °C, which has been shown to reduce energy use by 36% without compromising sample integrity [18]. Other ways to reduce energy consumption include turning off lights and equipment such as incubators when not in use, incorporating timers, closing fume hood sashes, using lower-power settings when possible, and replacing inefficient equipment. Additionally, transitioning mercury-based microscopy to LED increases energy use by >10-fold and is more economic, not requiring warm-up and cool-down periods [24]. Maintaining inventories will also allow laboratories to open freezers for less time and will produce less waste by keeping track of the expiration dates of products and adjusting procurement as needed. Disposing of old samples no longer needed and deleting old data can allow less physical and digital space utilization.

It is important to note the rapidly increasing carbon footprint of computational science, including artificial intelligence, although not as apparent and yet an emerging area of research. Computational science has an environmental impact not only from high-energy data centers/storages and high-performance software but also in forms such as electronic waste from hardware [25]. Thus, it is important to consider all sources of energy use in scientific work in sustainability efforts. Similar to waste audits, energy audits can be beneficial to identify areas of improvement and track progress and/or impact of changes.

## Catalyzing Cultural Changes In Laboratories And Institutions

Institutions are instrumental in catalyzing change and promoting sustainability within their community. Administrations and organization leaders should promote a culture of being environmentally conscious for widespread participation and long-term adherence to eco-friendly practices [6, 26]. Such institutional culture can be created by instituting a sustainability department as well as programs such as recycling pipelines, educational seminars, assistance in promoting eco-friendly practices and procurement, and events that incentivize sustainability (eg, challenges and competitions). Sustainability practices do in fact synergize with improving quality of work and patient care. Educational outreach programs can be effective in increasing awareness about the carbon footprints of laboratories, starting the conversation, and incentivizing groups to start their sustainability challenge. Institutions can also provide catered modules and training as well as catalogs for eco-friendly alternatives for laboratories to make information accessible.

Air travel for collaborative research, conferences, and meetings is another source of GHG emissions associated with laboratories. Institutions can play a role in catalyzing change on this front as travel and transport policies apply organization/institution-wide and not only to laboratory scientists and practitioners [27]. While engagement and interaction are valuable for scientific and collaborative practice in global health, scaling up local laboratory capacity in low- and middle-income countries (LMICs) settings serves to address equity and carbon costs [28]. Capacity strengthening of research laboratories in LMIC settings can better match the local needs of clinics, public health/surveillance, and research while maintaining meaningful partnerships and being cost and carbon saving when implemented sustainably. This in turn enables timely, appropriate, and evidence-informed local actions for outbreaks and/or endemic infectious diseases, thereby more widely improving global health equity and health security.

Catalyzing change in institutions and other organizations provides engagement and advocacy opportunities for individuals interested in sustainability work, such as forming or joining a greening task force that spearheads and monitors sustainability efforts. Individual laboratories can appoint a sustainability champion personnel. Other ways to contribute to sustainability efforts involve conducting research on sustainable solutions and innovations, advocating and creating programs within professional networks, and engaging in policy making. It is important to recognize the barriers that may arise in championing green practices in institutions and laboratories: time constraints, logistics, initial costs, availability of environmentally conscious alternatives, and compliance/resistance. To overcome these challenges, it is imperative to first emphasize the necessity and time-sensitive matter of the issue, hold a longer-term view, and establish sustainability as a priority for institutions and/or laboratories. Furthermore, regulations and incentives such as cost savings and rewards can be used to overcome resistance/inertia in adopting change. Normalizing and integrating green initiatives as part of regular workflow, instead of an “extra” step, can help to sustain the longevity of efforts. To overcome logistical challenges, laboratories and institutions can utilize many resources and frameworks that are available online and from other institutions that have been shown to make a significant impact, some of which are discussed in this article.

## GREENING CLINICAL MICROBIOLOGY LABORATORIES

The clinical microbiology laboratory performs high volumes of tests that guide patient care, infection prevention, and antimicrobial use. Due to the direct impact on patient care, laboratory testing is heavily regulated, and sterility and quality control are major focus points [29]. Additionally, the clinical laboratory is often a 24/7 operation, running multiple devices simultaneously for extended periods. Due to the need for single-use testing supplies, device usage, and biohazardous materials, the clinical microbiology laboratory is responsible for significant energy use and the production of billions of pounds of waste each year [30].

Concerning greening interventions, clinical laboratories can adopt practices similar to research laboratories. These actions include using glass materials rather than plastic whenever possible, turning off instruments when not in use, recycling, and working with suppliers to avoid products with excessive packaging [8, 30, 31]. In recent years, several studies have been published demonstrating the impact of green initiatives in clinical laboratories. In 2022, Chisholm et al described a carbon footprint reduction of at least 68.52 tons and anticipated cost savings of $43 000 per year due to implementing a greening program in the dermatopathology laboratory [32]. In addition to a positive environmental impact, the authors anticipate that they will experience less of an impact related to supply chain challenges, as well as cost savings associated with fewer reagent purchases through chemical recycling [31]. A 2023 study from the Vanderbilt University Medical Center retrospectively analyzed patient testing volumes and average weights for complete metabolic panel (CMP) testing kits to determine the amount of waste produced from performing CMPs routinely [33]. The CMP, identified as one of the top 10% of laboratory tests ordered at Vanderbilt, was associated with 1089.2 kg of reagent waste throughout the year. Of the total waste, approximately 21.4% was recyclable [33].

As previously mentioned, the most effective way to reduce waste is not to generate it at all [31]. While the clinical laboratory can implement numerous greening interventions, several other intervention points in the diagnostic process may be targeted for waste reduction and can reduce the number of unnecessary tests sent to the microbiology laboratory for testing.

## Diagnostic Stewardship

Diagnostic stewardship is an evolving process focused on ensuring that the right test is collected from the right patient at the right time [34]. In addition to potentially improving patient outcomes, diagnostic stewardship has been demonstrated to decrease unnecessary test and antimicrobial use and could aid in the reduction of waste produced by specimen collection, transport, processing, and testing [35]. The tests most commonly associated with overuse are blood, urine, and respiratory cultures, as well as stool *Clostridioides difficile* testing [34].

Although few studies have directly tied the impact of diagnostic stewardship interventions to waste, the potential benefit of such interventions is clear. For example, a multicenter quality improvement initiative focused on diagnostic stewardship of blood cultures in pediatrics resulted in a 33% relative reduction in blood culture use over 3 years [36]. Importantly, the rates of key clinical outcomes were similar after the implementation of the intervention, and patients were not harmed. Respiratory cultures are a significant driver of excessive antimicrobial use and require numerous laboratory resources [37–39]. For example, most respiratory cultures require multiple standard and specialized media plates for organism growth and subculturing; slides and related staining supplies for Gram staining; various biochemical reagents for organism identification; and reagents, supplies, and instrumentation for organism final identification and susceptibility testing. Because respiratory cultures often contain multiple organisms as well as multiple potential pathogens, the workup of these cultures can be extensive [40]. Reducing the use of respiratory cultures could significantly reduce laboratory waste, and this area is ripe for diagnostic stewardship intervention. In 2021, a clinical decision support intervention at Johns Hopkins University was implemented that focused on standardizing endotracheal aspirate culture (EAC) collection from pediatric patients undergoing mechanical ventilation. In the preintervention year, there were 557 EACs over 5092 ventilator days; after intervention implementation, there were 234 EACs over 3654 ventilator days. Overall, there was a 41% decrease in the monthly rate of EACs collected and sent to the microbiology laboratory for testing [39]. Additionally, there was an estimated cost savings of $26 000 per year and no negative impact on patient care.

Urinary tract infections are among the most common reasons why people seek medical care, accounting for 10 million health care visits annually [41]. Urine cultures are frequently ordered, often inappropriately, and each culture is associated with plastic collection cups and laboratory testing supplies. Recent diagnostic stewardship studies have demonstrated significant decreases in test use through quality improvement interventions involving clinical decision support tools, test-ordering requirements in the preanalytic period, and rejection criteria for testing in the analytic period [41, 42]. While studies have not tied decreases in urine testing directly to laboratory waste, it is possible to imagine the significant impact of these decreases on waste, particularly given the frequency of urinary tract infection testing each year.

Microbiology laboratories may identify other areas of possible waste reduction by reviewing frequently repeated tests and using existing laboratory data to assess whether repeat testing is necessary and could be mitigated. In a recent Erasmus University Medical Center study in the Netherlands, medical microbiologists reviewed >18 000 successive antimicrobial susceptibility test results to determine the risk of organisms becoming resistant to tested antibiotics within various periods [43]. Currently, most medical microbiology laboratories will repeat an antimicrobial susceptibility test within 7 days of initial testing to ensure that the organism's susceptibility profile has not changed. However, the authors of this study found that the risk was low at their center and that it would be feasible to omit follow-up testing within 7 days. Ultimately, they concluded that this modification will save money and time and reduce laboratory waste [43].

There are many opportunities for the clinical microbiology laboratory to reduce waste and environmental footprint: recycling practices, plastic reduction, and working with vendors committed to environmentally friendly practices. Notably, diagnostic stewardship projects may serve as impactful interventions in waste reduction. Microbiology laboratories should work closely with clinical partners to identify areas of optimal intervention and ensure that data on waste reduction, as well as the impact on clinical outcomes, are collected.

## GREENING UP VETERINARY DIAGNOSTIC LABORATORIES, CLINICS, AND ONE HEALTH

**Figure 1.**
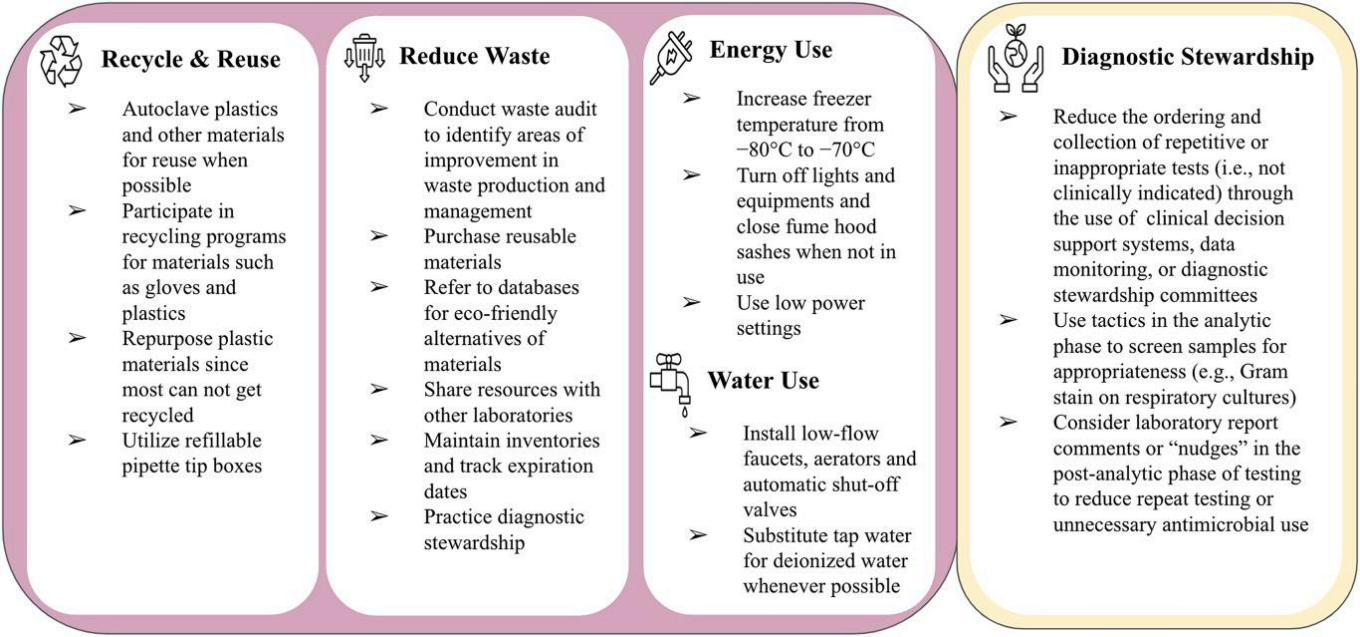
Eco-friendly practices that laboratories can adopt to address waste production and energy and water use.

One Health highlights the interrelationships among human, animal, and environmental health and helps to understand and minimize the impact of the transmission of zoonotic diseases [44]. Zoonotic pathogens are responsible for >60% of the infectious diseases in humans and have the potential to result in morbidity and mortality [44–46]. As public health professionals, veterinarians are in a unique position to contribute solutions to One Health, planetary health, and climate change. Laboratory facilities for animal health play a crucial role in the diagnosis and surveillance of veterinary diseases, with the objective of preventing and controlling transboundary animal diseases and zoonotic infections and helping to produce large amounts of data for animal and public health [47]. Veterinary clinics, hospitals, and laboratories also have a role and responsibility in implementing environmentally friendly practices. Veterinary waste management techniques exhibit substantial synergy among many industries, such as agriculture, laboratories, and pharmaceutical businesses involved in the handling of various waste categories [48]. As in research and microbiology laboratories, veterinary hospitals undoubtedly have significant environmental impacts and generate excessive waste, including phlebotomy supplies, packaging, and biohazardous waste such as needles, surgical waste, and animal carcasses. Despite the importance of the subject, there is limited climate change and sustainability teaching in veterinary curriculums [49, 50]. To mitigate their environmental footprint, veterinary clinics can segregate waste, utilize reusable equipment and surgical textiles, purchase items with minimal packaging, minimize pharmaceutical waste, utilize sterilization, and adopt rechargeable batteries for mobile anesthetic units. The ACT database can be used to select environment-friendly items and should be accessible to veterinary consumers to enable sustainable purchasing choices [19]. Additionally, incorporating veterinary telemedicine into health care practice can reduce emissions associated with transportation, and digitization of medical records and invoices will minimize paper waste. Waste production can also be reduced in daily clinical activities such as cleaning, treatments, and surgery by using less. A summary of eco-friendly practices for all laboratories can be found on Figure 1. Veterinary professional organizations should enhance their policies, providing more education on policy and advocacy for veterinary professionals and students and launching actions to address climate change and planetary health concerns [48, 51].

## GLOBAL PERSPECTIVE: A CASE STUDY ON HEALTH CARE WASTE MANAGEMENT IN 2 RURAL HOSPITALS IN KENYA

### Waste Management

Solid waste mismanagement is a worldwide issue in terms of environmental contamination, social inclusion, and economic sustainability [52, 53]. Poor hospital waste management poses a risk of exposure to and subsequent transmission of infections; results in pollution of air, water, and land; and significantly contributes to adverse effects on human, animal, and environmental health [54]. Multiple studies suggest that medical waste management in the African continent is still in its infancy, without the ability to tackle the quantity of medical waste produced in light of an increasing population and limited resources [55–57]. Much of the waste is dumped without treatment in open dumps [58]. Incinerators are also limited and, where available, are broken down, improperly constructed, or not being used. This section presents an observational study of health care waste management systems in 2 rural health care facilities in Kenya to provide an overview of medical waste, including its sources and categories to identify current practices and areas that require improvement.

Port Victoria and Sio Port Hospitals are level IV subcounty rural hospitals in Busia County, Kenya. Waste generated from the different hospital departments (solid or liquid) is categorized as infectious (hazardous) or noninfectious (nonhazardous). Among the waste generated from the wards are hypodermic needles, diapers, blood-soaked cotton wool, gynecologic items, and gloves. The process of segregating, disinfecting, and transporting waste is a standardized practice implemented across hospital departments. The hospital staff segregates waste in the department in which it was generated. For instance, nurses or doctors at the bedside, in the ward, at the causality area, or in the operating theater segregate waste into designated bins. However, limited funding in most LMIC hospitals does not allow for contractual services, such as medical waste removal, transportation, and storage. This is left to the improperly trained sanitary disposal workers of each facility to address.

At these hospitals, waste segregation is organized into 4 categories: noninfectious, infectious, highly infectious, and sharps. Proper segregation is achieved by the use of colored containers to effectively separate infectious waste [59]. The color coding includes black bins for noninfectious material; yellow for infectious waste to be treated with 0.5% hypochlorite solution before disposal into burning chambers or incinerator; and red for anatomic and pathologic waste, also treated with 0.5% hypochlorite solution before disposal into the incinerator by a trained professional. Color code is an easy method for identification to ensure safe handling, transportation, and waste treatment. Red and yellow bin wastes are discarded daily due to their infectious effects. The waste is decontaminated, tightly tied, and transported on a wheelbarrow to the incinerator. A sharps container is used for cannulas, broken slides, scalpels, needles, lancets, and syringes. This bin is used until it is three-quarters full, where it is sealed appropriately and transported to a central place in the hospital. On a monthly basis, the sharps containers are transported to Busia County Referral Hospital (level V), where they are incinerated. Expired pharmaceuticals and large quantities of obsolete drugs in wards or departments are returned to the pharmacy for disposal.

## Transportation Of Hospital Wastes

Transportation of hospital waste is done either on-site or off-site. On-site involves the transfer of waste from the source of generation to internal storage facilities, while off-site involves the transfer of waste from internal storage facilities to the final disposal site. On-site transportation is usually performed by means of wheeled trolleys, containers, or wheelbarrows that are not used for any other purpose, while off-site is usually by means of vehicles. The facilities have temporary storage that is secured before transportation for disposal. The area for burning waste also has to be secured to prevent animals and birds from accessing the waste.

## Impact Of Waste Management On Local Communities In Kenya And Global South

Biomedical waste in Kenya and the Global South is commonly disposed of in open dump sites or informal landfills, which often lack proper management and infrastructure. These are typically located in the outskirts of urban areas, where they not only contribute to climate change globally but pose numerous environmental and public health hazards locally: air and water pollution, land degradation, methane and hazardous leachate emission, methane explosions, unpleasant odors, attraction of disease-carrying animals, illegal dumping, and littering [60]. Currently, the country is experiencing serious challenges in waste collection, disposal, and recycling. These challenges have resulted in environmental degradation, public health hazards, and economic losses. Addressing these challenges calls for a multifaceted approach that includes increasing investment in waste management infrastructure and services, strengthening regulations and enforcement, promoting public awareness and education, and encouraging the participation of the private sector and civil society in waste management.

In summary, we observed that greening initiatives such as reducing, reusing, and recycling waste, as well as strict waste segregation, are inadequate in these hospitals in Kenya and likely elsewhere in LMICs. Sustainable waste management requires a multifaceted approach—specifically, improving leadership on waste management, promoting education to engage all staff in sustainable health care practices, rectifying misconceptions regarding infectious and/or safety risks and concerns about increased workload, and facilitating positive staff attitudes and acceptance of change.

## CONCLUSION

Health care and research personnel, as well as institutions and supply industries, have an obligation to minimize waste production, environmental pollution, and unsustainable waste disposal practices that cause direct and indirect harm to planetary health. Sustainable practices in laboratories are not only good for the environment but also economical, and they save time for laboratory personnel. Not only do current waste practices within health care and scientific research exacerbate local and global inequities, but they also contribute to environmental pollution, with enormous plastic waste production and GHG emissions resulting in significant increases in infectious disease transmission and other harms to human and planetary health. Sustainable practices in waste management, reducing waste production, and greening up laboratories provide ample health, equity, and economic benefits to all and can advance global equity. Additionally, eco-friendly practices do not necessarily require additional time and resources, especially if supported by institutional leadership and culture, nor do they compromise patient care. Institutions can benefit from significant cost savings from greening up efforts. Behavioral changes may be challenging to implement at first, but with institutional and individual investment, even small changes can produce tremendous results toward a greener planet and healthier people.

## Note

***Potential conflicts of interest*.** All authors: No reported conflicts. All authors have submitted the ICMJE Form for Disclosure of Potential Conflicts of Interest.
